# Comprehensive surgery of complex scalp arteriovenous fistula: a successful trial

**DOI:** 10.3389/fsurg.2024.1492616

**Published:** 2025-01-13

**Authors:** Zhiming Ma, Zhihao Zou, Dajiang Xie, Linlin Qian

**Affiliations:** ^1^ Department of Neurosurgery, General Hospital of Xinjiang Military Command, Urumqi, China; ^2^ Department of Neurosurgery, Sir Run Run Shaw Hospital, College of Medical Sciences, Zhejiang University, Hangzhou, China

**Keywords:** scalp arteriovenous fistula (S-AVF), comprehensive approach, endovascular embolization, surgical resection, trauma

## Abstract

**Background:**

Traumatic scalp arteriovenous fistula is a rare vascular abnormality. Open surgical removal and embolization have been employed to address this condition.

**Methods:**

In this report, we present a case involving a 41-year-old man who exhibited a progressively enlarging pulsatile mass in his right occipital scalp. Computerized tomography angiography (CTA) and digital subtraction angiography (DSA) identified a complex scalp arteriovenous fistula (S-AVF). The combination of clinical symptoms and neuroimaging findings facilitated the diagnosis.

**Results:**

Following a thorough discussion, we implemented a comprehensive strategy that included both endovascular embolization and surgical resection. The patient demonstrated an excellent prognosis with no reported discomfort.

**Conclusions:**

A comprehensive surgical approach should be considered in the management of patients with complex scalp arteriovenous fistula.

## Introduction

Traumatic scalp arteriovenous fistula (S-AVF) is a rare vascular abnormality (1, 2). Since the original report by Wardrop in 1827, only a limited number of cases have been documented (3). This condition is characterized by a direct connection between the arterial feeding vessels of the scalp and the draining veins, bypassing the intervening capillary beds (2). In contrast, arteriovenous malformation (AVM) is a congenital lesion that features a nidus between the arterial and venous systems (4–6). Due to abnormal hemodynamics, S-AVF can progress from a small swelling to a palpable subcutaneous mass, presenting various symptoms such as headache, bruits, tinnitus, epilepsy, hemorrhage, and scalp necrosis (7, 8). Several primary treatment methods, including open surgical removal and embolization, have been employed to address this condition (6, 9, 10). In this report, we present a distinct case of a ruptured Yokouchi type C S-AVF, which was successfully diagnosed through preoperative symptoms and neuroimaging studies, and resolved via a comprehensive treatment approach that included endovascular embolization and surgical resection. The patient exhibited an excellent response, suggesting that comprehensive treatment may be clinically beneficial for complex S-AVF.

## Clinical report

### History and examination

**Figure 1:**
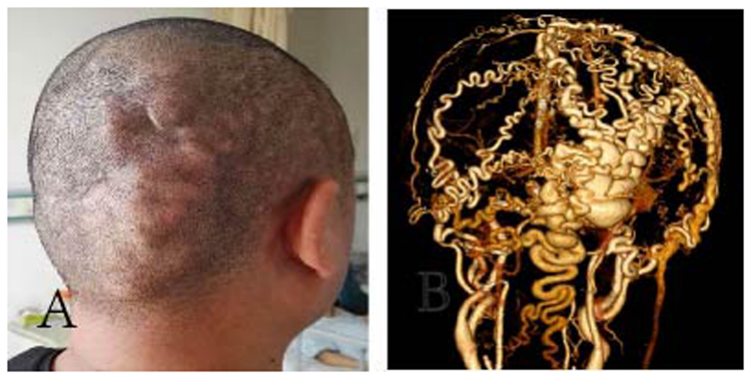
Scalp arteriovenous fistula (S-AVF). **(A)** 41-year-old man presented with a pulsating mass measuring 12 × 7 × 3 cm in size in his right occipital part. **(B)** CTA shows a complex multiple arteriovenous fistula located in the right occipital scalp and part of the right temporoparietal region, blood was supplied by branches of bilateral external carotid arteries and drained by the right superficial occipital vein.

**Figure 2:**
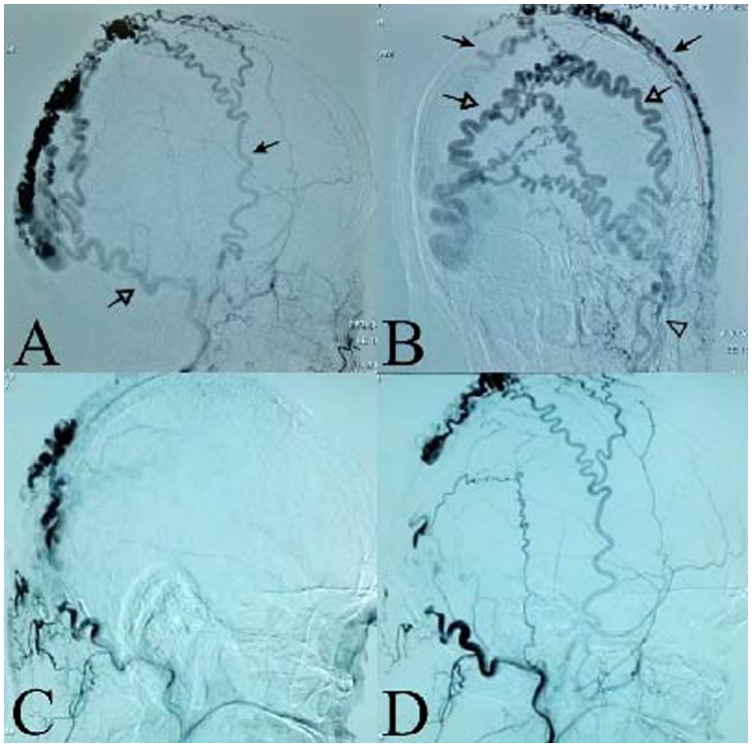
Interventional embolization of S-AVF. **(A)** Right external carotid artery angiography. Black arrow shows the right superficial temporal arteries (STA); white arrow shows the right occipital artery (OA). **(B)** Left external carotid artery angiography. The black arrow shows the bilateral superficial temporal arteries (STA); the white arrow shows the bilateral occipital arteries (OA). (C/D) DSA of right occipital artery embolization demonstrated the partial occlusion of the S-AVF.

A 41-year-old man presented with a progressive, painless, pulsatile mass in his right occipital skull (Figure 1). He exhibited no neurological deficits. The patient reported having accidentally bumped his head against a corner of a window approximately 20 years prior. A general examination revealed a pulsating mass measuring 12 cm × 7 cm, accompanied by bilateral tortuous vessels. A loud bruit was detected upon auscultation. Computed tomography angiography (CTA) revealed a complex arteriovenous fistula located in the right occipital scalp and extending into part of the right temporoparietal region (Figure 1). Digital subtraction angiography (DSA) confirmed that the feeding arteries included the bilateral superficial temporal arteries (STA), bilateral occipital arteries (OA), and the right posterior auricular artery (PAA). The dominant draining vein was identified as the right superficial occipital vein. Furthermore, there was no connection observed between the intracranial and extracranial vessels (Figure 2A,B).

### Operation and postoperative course

**Figure 3:**
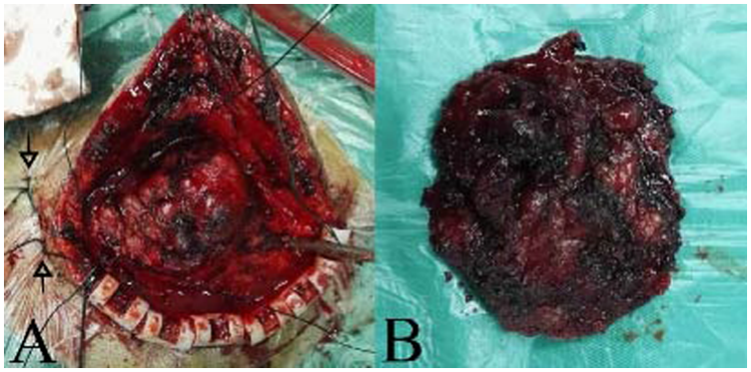
The mass of surgical resection. **(A)** The white arrows show suture lines ligating the feeding arteries near the mass. **(B)** The mass was totally removed.

**Figure 4:**
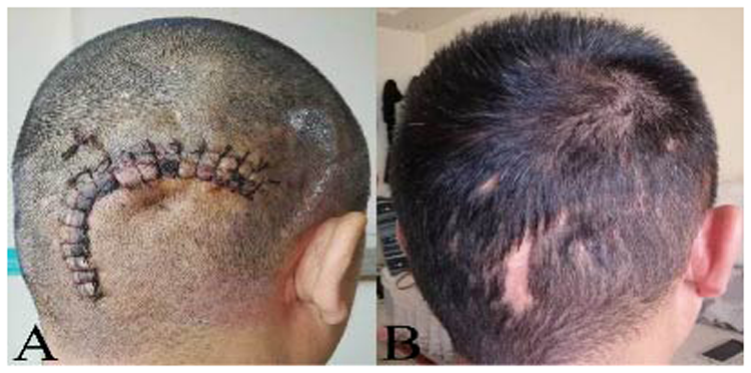
The scalp after surgical resection. **(A)** The surgical incision was dry and clean, and the raised mass disappeared on the third day. **(B)** After the 12-month follow-up, no recurrence and nothing abnormal was observed, except for lack of hair in part of surgical incision.

After analyzing the angio-architecture and size of the S-AVF, we decided to proceed with a comprehensive treatment involving endovascular embolization and surgical removal. Onyx liquid embolic material was successfully injected into the branches of the right occipital arteries supplying the S-AVF. Post-embolization digital subtraction angiography (DSA) revealed partial occlusion of the S-AVF, with the bilateral superficial temporal arteries only faintly contributing to its perfusion (Figure 2C,D). To prevent scalp necrosis, we ceased endovascular treatment at this point. Notably, the mass size was significantly reduced compared to pre-embolization measurements. Following several days of observation, both the temperature and color of the skin returned to normal. Surgical resection was then performed in a prone position under general anesthesia. A semicircular scalp incision was made along the mass, and to minimize bleeding, we initially ligated the tortuous vessels surrounding the mass. Upon incising the scalp, we observed an abundance of large, tortuous, dilated venous plexuses situated beneath the galea aponeurotica. We ligated and completely excised the feeding arteries and draining veins. Finally, we removed the mass and sutures, closed the flap in a standard manner (Figure 3, Figure 4A).

Histopathological examination confirmed the presence of an arteriovenous fistula. Postoperatively, all symptoms resolved, and no complications arose. At the 12-month follow-up, no recurrence was found (Figure 4B).

## Discussion

### The diagnosis of S-AVF

Traumatic S-AVF is an uncommon vascular disease, reported infrequently in the literature (11–13). This condition is characterized by a direct connection between the arterial feeding vessels and the draining veins of the scalp, bypassing the intervening capillary beds (2). The superficial temporal artery (STA) is most commonly involved due to its long and relatively superficial course in the scalp (2, 14). Treatment is challenging due to the complex pathogenesis and heterogeneous angio-architecture associated with the disease.

The main symptoms of traumatic S-AVF can vary and may include a pulsatile mass, bruits, tinnitus, headaches, epilepsy, hemorrhage, and scalp necrosis (7, 8). Sofela et al. reported that congestive heart failure may even occur in severe cases (8).

The pathophysiology of traumatic S-AVF remains not fully understood. The laceration theory posits that simultaneous laceration of both the artery and the adjacent vein leads to the formation of the fistula (15). An alternative mechanism, known as the disruption theory, suggests that the rupture of the vasa vasorum in the artery wall initiates the process. This rupture leads to the proliferation of endothelial cells from the damaged vasa vasorum, facilitating the formation of numerous small vessels and resulting in vascular communication channels between the artery and vein (1).

Yokouchi classified S-AVF into three types: Type A, a single fistula fed by a single proximal feeding artery; Type B, a single fistula fed by multiple feeding arteries; and Type C, multiple fistulas with plexiform feeding arteries and a main dilated draining vein (16). Our case falls under Type C, which is considered to require a comprehensive treatment approach that combines embolization with surgical removal (16, 17).

The diagnosis of S-AVF is based on local symptoms and characteristic AVF manifestations observed through imaging techniques such as magnetic resonance imaging/angiography (MRI/MRA), computed tomography angiography (CTA), and digital subtraction angiography (DSA). Notably, DSA is regarded as the gold standard for imaging S-AVF, as it allows for dynamic observation of both the feeding arteries and the draining veins.

### The management of S-AVF

The goal of treating S-AVF is to prevent hemorrhage and scalp necrosis, as well as to alleviate the pulsatile mass. Traditionally, open surgery has been the standard treatment, offering clear removal of lesions with a low incidence of complications (18). However, with the advent of micro-catheters and enhanced embolization materials, endovascular therapy has emerged as a significant therapeutic option for S-AVF. Nonetheless, endovascular treatment for complex multiple S-AVF can be challenging, as it carries a risk of recurrence post-embolization and may not adequately address all abnormal vessels, thus failing to achieve a radical cure for the mass (19). Additionally, complications following embolization can include tenderness, hyperemia, scalp necrosis, and the potential escape of embolic materials into the circulatory system (2, 20), as well as thrombosis formation or vessel dissection (21).

In our current case study, we present a comprehensive approach to managing complex multiple S-AVF (Yokouchi Type C) through a combination of endovascular embolization and surgical removal. Given the definitive preoperative diagnosis and the tailored therapeutic plan for S-AVF, embolization was frequently employed as an initial strategy to mitigate the risks associated with surgical resection. We emphasize the importance of monitoring the skin's temperature and color post-embolization to detect signs of ischemia or necrosis, which may necessitate flap transfer during open surgery, alongside the administration of drugs to enhance microcirculation. Following embolization, the mass size significantly decreases, and substantial blood loss is reduced during surgical resection. Clinical practice has demonstrated that ligating the feeding arteries adjacent to the mass should be prioritized to minimize bleeding during surgery.

This case contributes to our understanding of this rare condition. The combination of clinical symptoms and neuroimaging studies plays a crucial role in diagnosis. The prognosis for complex multiple S-AVF is excellent following a comprehensive surgical approach.

## Conclusions

Traumatic scalp arteriovenous fistula (S-AVF) is a rare vascular abnormality characterized by a direct connection between the arterial feeding vessels and the draining veins of the scalp, effectively bypassing the intervening capillary beds. Comprehensive treatment methods, including open surgical removal and endovascular embolization, have been employed to address this condition. Overall, the prognosis is generally favorable following comprehensive treatment.

## Data Availability

The data presented in this article is available upon request from the corresponding author.
